# Determinants of severe acute malnutrition among under-five children in Ethiopia: analysis using data from the 2016 Ethiopia Demographic and Health Survey

**DOI:** 10.3389/fnut.2024.1403591

**Published:** 2024-08-15

**Authors:** Abriham Shiferaw Areba, Desta Erkalo Abame, Lire Lemma Tirore, Bisrat Feleke Bubamo

**Affiliations:** ^1^School of Public Health, College of Medicine and Health Sciences, Wachemo University, Hosanna, Ethiopia; ^2^Department of Health Informatics, College of Medicine and Health Sciences, Wachemo University, Hosanna, Ethiopia; ^3^Department of Public Health, College of Medicine and Health Sciences, Wachemo University, Hosanna, Ethiopia

**Keywords:** severe acute malnutrition, under-five children, Demographic and Health Survey, Ethiopia, malnutrition

## Abstract

**Objective:**

Malnutrition is a silent killer that is under-reported, under-addressed, and, as a result, emphasized. This study aimed to identify the determinants of severe acute malnutrition (SAM) among under-five children in Ethiopia.

**Methods:**

Cross-sectional data from the 2016 Ethiopian Demographic and Health Survey (EDHS) were used. A total of 6,170 under-five children were included in the current analysis. The data were cleaned and analyzed using STATA 14. An adjusted odds ratio (AOR) and their 95% confidence intervals (CIs) were calculated to determine the association between factors and outcomes. A *p*-value of less than 0.05 was considered significant in multivariable logistic regression.

**Results:**

A multivariable logistic regression revealed that under-five children with the age of children in months 6–11 (AOR = 1.52, 95% CI: 1.25, 1.86), 12–23 (AOR = 1.98, 95% CI: 1.65, 2.37), and 24–59 months (AOR = 1.71, 95% CI: 1.40, 2.08), birth order between fourth and fifth (AOR = 1.24, 95% CI: 1.01, 1.54), having fever (AOR = 1.31, 95% CI: 1.09, 1.58), anemic children (AOR = 1.21, 95% CI: 1.07, 1.36), age of mothers in years 25–34 (AOR = 0.60, 95% CI: 0.51, 0.72) and 35–49 (AOR = 0.49, 95% CI: 0.39, 0.63), antenatal care (ANC) visits (AOR = 0.83, 95% CI: 0.71, 0.92), rural residence (AOR = 2.98, 95% CI: 2.54, 3.49), and solid fuels users (AOR = 2.46, 95% CI: 1.86, 3.26) were significant predictors.

**Conclusion:**

Older age of children, those with higher birth order, those having fever, anemic children, those living in rural areas, and solid fuel users were more likely to suffer from SAM, while older mothers and those having ANC visits had reduced SAM as significant predictors.

## Introduction

Globally, child malnutrition is a public health problem with major consequences for child survival, damaging the cognitive and physical development of children and the economic productivity of individuals’ strengths and societies (1). Child malnutrition is one of the measures of health status that the World Health Organization (WHO) recommends for equity in health (2). A weight-for-height ratio of less than minus 3 standard deviations below the median reference population, a weight-for-height ratio of less than 70%, or mid-upper arm circumference (MUAC) of <110 mm or the occurrence of nutritional edema are all indicators of SAM (3). Acute malnutrition affects 52 million (or 8.3%) under-five children around the world today. The bulk of the children affected live in South and Southeast Asia and Sub-Saharan Africa, accounting for more than 90% of the total. Contrary to popular opinion, acute malnutrition (also known as wasting) does not only occur in emergencies; it also occurs frequently in healthy settings in countries such as India, Indonesia, Kenya, and Ethiopia (4).

Among sub-Saharan countries, the prevalence of wasting in Ethiopia is 10%; within Ethiopia, there is a regional variation in wasting. Amhara, Benishangul-Gumuz, Afar, and Dire Dawa are the most affected by child stunting (41–46%), whereas wasting is the highest in Somali (23%), Afar (18%), and Gambella (14%). The reason may be that hard-to-reach areas of Afar, Somalia, Benishangul-Gumuz, and Gambella were characterized by drought-related public health and nutrition problems (5). The prevalence of malnutrition ranges from 1.4% in Addis Ababa to 10% in Somalia, with a further distinction of a higher prevalence of severe malnourishment (6).

SAM accounts for 21% of disability-adjusted life years in under-five children. SAM has both immediate and long-term nutritional implications, including a lower intelligence quotient (IQ) and stunted growth (7). Malnutrition often impairs the immune system’s ability to function, making children more vulnerable to other diseases. Diarrhea, pneumonia, measles, and malaria morbidity all raise the risk of death in malnourished infants. According to a study from Ethiopia, 24.3% of children with SAM developed pneumonia, and many more (21%) developed diarrhea and tuberculosis (11%). Vitamin A and zinc shortages are also thought to be responsible for 0.6 million and 0.4 million deaths, respectively (8).

Approximately 85% of severely wasted children with no medical problems can be treated at home in outpatient care, eliminating the need for hospitalization. This has the benefit of shielding them from infection while also allowing mothers to care for the rest of the family while caring for the malnourished infant (9). Outpatient therapeutic feeding services (OTPs) are given at health facilities in Ethiopia as part of routine health care. Community health workers (CHWs) deliver ready-to-use therapeutic foods (RUTFs) to be consumed at home once a week, as well as a course of regular drugs such as amoxicillin, folic acid, vitamin A, measles vaccine, and deworming (10). Malnutrition in children is caused by a lack of food, diarrhea, other diseases, poor hygiene, and a lack of parental education (11).

According to the literature, the mother’s age at birth, the child’s age, low socioeconomic status, family educational level, birth interval, family size, breastfeeding, diarrheal diseases, febrile illnesses, vaccination status, and initiation of complementary feeding at the age of 6 months were all important determinants of SAM in under-five children (12–15).

Although there is a continually high magnitude of severe acute child malnutrition in Ethiopia, published research does not give significant evidence on its risk factors in all parts of the country. Most surveys conducted in Ethiopia had a lesser number of research participants and were not conducted on a large scale, making them ineffective for identifying risk factors. In this research, it was observed that SAM was significantly associated with household and community characteristics such as ANC visits, the anemic status of the mother, rural dwellings, and types of fuel used for cooking. As a result, this study was carried out to identify determinants of SAM among under-five children in Ethiopia from the 2016 EDHS. In general, the motivation behind this study is to address the following major research questions:

What is the prevalence of SAM among under-five children in Ethiopia?

What are the key risk factors associated with severe acute malnutrition among under-five children in Ethiopia?

## Methods

### Study area and period

The data for this analysis originated from the 2016 EDHS, which was obtained from Ethiopia’s nine regions and two administrative cities between 18 January and 27 June 2016. Ethiopia, located in the Horn of Africa, is a multilingual and multicultural country. Eritrea, Djibouti, Somalia, Kenya, South Sudan, and Sudan are its neighbors. The nation covers 1.1 million square kilometers and has elevations ranging from 4,620 m above sea level at Ras Dashen Mountain to 148 m below sea level at the Danakil Depression (16).

### Study design

A cross-sectional study design was conducted.

### Data source

Ethiopian Demographic and Health Survey 2016, which was conducted by the Central Statistical Agency with the help of the Ministry of Health (MoH), every 5 years timely estimates of key demographic and health indicators with the MoH’s request and this is the fourth round (17). The data were requested by sending the proposal’s title and summary. The data secondary sets were obtained from the Department of Homeland Security’s website.^1^

### Source population

The source populations were all under-five children in Ethiopia.

### Study population

The study population was all under-five children in the 2016 EDHS.

### Weighting the data

To “take into account” or “adjust for” disproportionate sampling and non-response, Demographic and Health Surveys (DHS), weights the data. Weights were used to restore the representativeness of the sample, so the total sample distribution “looks like” the country’s actual population distribution. The sampling weight variable was created using DHS’s suggestion that sample weight (V005) be divided by one million (18).

### Variables in the study

#### Outcome variable

The outcome variable was SAM, standing among under-five children as outlined by weight-for-height < − 3z scores relative to WHO standards (19). SAM of the i^th^ child was coded as binary variables based on the standard definitions.


$$Y_{i}=\{\begin{array}{c} \begin{array}{l} 1\;\mathrm{Severe}\;\mathrm{acute}\;\mathrm{malnutrition}\;\mathrm{if}\;z\;\mathrm{score}<-3SD\;\mathrm{from}\;\\ \mathrm{the}\;\mathrm{median}\;\mathrm{of}\;\mathrm{the}\;\mathrm{WHO}\;\mathrm{standards} \end{array}\\ \begin{array}{l} 0\;\mathrm{Normal}\;\mathrm{if}\;z\;\mathrm{score}>-3SD\;\mathrm{from}\;\mathrm{the}\;\\ \mathrm{median}\;\mathrm{of}\;\mathrm{the}\;\mathrm{WHO}\;\mathrm{standards} \end{array} \end{array}$$

Y_i_ = represents the SAM of the i^th^ child.

#### Predictor variables

To analyze the determinants of SAM among under-five children, the study considered the following characteristics as independent variables after reviewing different literature: the age of children was the main exposure variable of interest. WHO recommends for children less than 2 years which includes 0–5 months we measure the recumbent length and then we measured weight, so we assessed their weight for length (weight-for-length) (20, 21). The household was explored for the child’s age by interviewing the mothers with the questions “If mother interviewed: Copy child’s date of birth (day, month, and year) and age from birth history. If the mother is not interviewed, ask: what is (name)‘s date of birth and age? To check child born in 2003–2008?” The options for the response were between 0 and 59 months or (0–59 months). Hence, the above-mentioned age of children was categorized as 0–5 months, 6–11 months, 12–23 months, and 24–59 months (5, 22, 23), sex of children (male, female).

Birth order refers to the order in which a child is born in their family (24). It is categorized by first, second and third; fourth and fifth; and sixth and above (5, 25). Breastfeeding is the process of providing human breast milk to a child. Breast milk can be expressed directly from the breast, by hand, or pumped and supplied to the newborn (26). Breastfeeding categorized by no or yes (27), Cough (no, yes), anemia is a medical disorder in which the red blood cell count or hemoglobin level is lower than normal. Anemia is usually defined in men as a hemoglobin level of less than 13.5 g/100 mL and in women as a hemoglobin level of less than 12.0 g/100 mL (28), which is categorized as (not anemic, anemic) (27). Fever: refers to mothers/caretakers thought as the child experienced an unusual increase in temperature for 2 weeks before the survey, categorized as no or yes (6), ANC visits (no, yes), work status of mother (no, yes), residence (urban, rural), age of mother (15–24, 25–34 and 35–49 Years).

Based on the 2016 EDHS, the sources of drinking water and toilet facilities were recorded as either improved or unimproved and the type of cooking fuel was also recorded as a cleaner fuel or solid fuel. An improved water source includes piped water, public taps, standpipes, tube wells, boreholes, protected dug wells and springs, and rainwater, which is considered an improved water source. Unimproved sources include unprotected dug wells and springs, tanker trucks/carts with a small tank, and surface water. Improved toilet facilities include any non-shared toilet, flush/pour-flush toilets to piped sewer systems, septic tanks, pit latrines, ventilated improved pit (VIP) latrines, pit latrines with slabs, and composting toilets. The unimproved facility includes a shared toilet, flush/pour-flush not to sewer/septic tank/ pit latrine, pit latrine without slab/open pit, and hanging toilet/hanging latrine. For household cooking fuel types, electricity, liquefied petroleum gas, natural gas, biogas, and kerosene are cleaner fuels and coal/lignite, charcoal, wood, straw/shrubs/grass, agricultural crops, and animal dung are solid fuel (29).

#### Eligibility criteria

Inclusion criteria: all under-five children who were malnourished.

**Figure 1:**
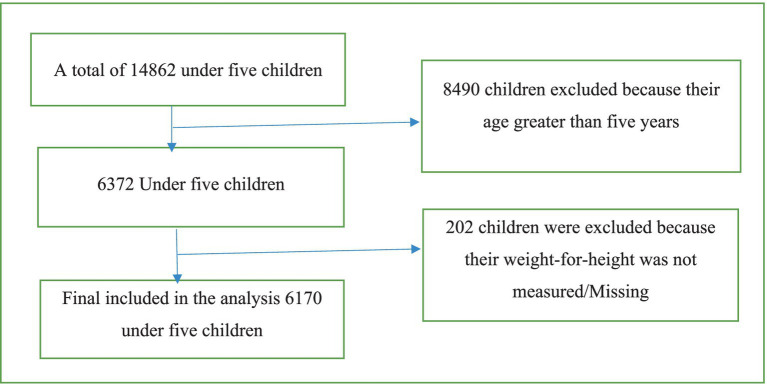
Included and excluded cases for SAM among under-five children in Ethiopia.

Exclusion criteria: children with missing values and an age greater than 5 were excluded (Figure 1).

### Statistical analysis

We have used STATA analysis software version 14 for data analysis. Descriptive statistics were used to report the distribution of the data among variables using frequency and percentage. Tables and figures were used for data presentation. A binary logistic regression model was fitted and was used to determine whether there is an association between independent variables and SAM in children. Variables with a *p*-value of <0.20 were entered into a multivariable model to determine the association between the independent variables and SAM while adjusting for other potentially confounding variables. For multivariate analysis, statistical significance was determined using a 95% CI and a p-value of less than 0.05. As a consequence, the backward exclusion is used to omit non-significant variables from the final model (30).

## Results

When we look at the children’s ages, we find that 908 (14.72%) of the children are between the age groups 6 and 11 months, which is the lowest number of children found, and 2,620 children are between the ages of 22 and 59 months, which is the highest number of children found (42.46%). Women’s residence is split into two categories: urban and rural, with 1,248 (20.23%) residing in urban areas and 4,922 (79.77%) living in rural areas (Table 1).

**Table 1:** Percent distribution and bivariable analysis for SAM among under-five children Ethiopia, EDHS 2016.

Variables	Categories	Frequency (%)	COR[95% CI COR]	*p*-value
Age of children	0–5 Months	988 (16.01)		
	6–11 Months	908 (14.72)	1.46 (1.21, 1.75)	0.001**
	12–23 Months	1,654 (26.81)	1.82 (1.55, 2.15)	0.001**
	24–59 Months	2,620 (42.46)	1.55 (1.33, 1.80)	0.001**
Sex of children	Male	3,193 (51.75)		
	Female	2,977 (48.25)	0.98 (0.89, 1.09)	0.76
Birth order	First	1,190 (19.29)		
	Second and third	1933 (31.33)	0.97 (0.84, 1.13)	0.72
	Fourth and fifth	1,428 (23.14)	1.24 (1.06, 1.46)	0.009*
	Sixth and above	1,619 (26.24)	1.05 (0.89, 1.22)	0.568
Breastfeeding	No	2,311 (37.46)		
	Yes	3,859 (62.54)	0.932 (0.84, 1.04)	0.204
Cough	No	5,043 (81.73)		
	Yes	1,127 (18.27)	1.11 (0.97, 1.27)	0.143*
Fever	No	5,157 (83.58)		
	Yes	1,013 (16.42)	1.273 (1.10, 1.47)	0.001**
Anemia status	Not Anemic	4,147 (67.21)		
	Anemic	2023 (32.79)	1.38 (1.23, 1.55)	0.001**
Age of mother	15–24 years	1,552 (25.15)		
	25–34 years	3,076 (49.85)	0.63 (0.55, 0.72)	0.001**
	35–49 years	1,542 (24.99)	0.59 (0.51, 0.69)	0.001**
ANC visits	No	2051 (33.24)		
	Yes	4,119 (66.76)	0.59 (0.52, 0.66)	0.001**
Work status of the mother	No	3,495 (56.65)		
	Yes	2,675 (43.35)	0.86 (0.77, 0.95)	0.004**
Place of Residence	Urban	1,248 (20.23)		
	Rural	4,922 (79.77)	3.94 (3.46, 4.48)	0.001**
Source of drinking water	Unimproved	2,292 (37.15)		
	Improved	3,878 (62.85)	0.65 (0.58, 0.73)	0.001**
Types of cooking fuel	Cleaner fuel	351 (5.69)		
	Solid fuel	5,819 (94.31)	6.25 (4.88, 8.00)	0.001**
Types of toilet facility	Unimproved	5,892 (95.49)		
	Improved	278 (4.51)	0.33 (0.26, 0.42)	0.001**

CI, Confidence interval; COR, crude odd ratio; **p < 0.01, *p < 0.05.

Age of children, birth order, cough in last 2 weeks, fever in last 2 weeks, anemia status, age of mother, ANC visits, work status of the mother, place of residence, source of drinking water, type of cooking fuel, and type of toilet facility were associated with SAM in the bivariate logistic regression analysis, but the sex of the children and breastfeeding were not significant at the modest level of significance at a *p*-value of 0.2 (Table 1).

At a 5% level of significance, the age of children, birth order, fever in the previous 2 weeks, anemia status, age of mother, ANC visits, place of residence, and type of cooking fuel had significant effects on SAM. When other variables were kept constant in multivariable regression, children in the age group 6–11 months (AOR = 1.52, 95% CI: 1.25, 1.86), 12–23 months (AOR = 1.98, 95% CI: 1.65, 2.37), and 24–59 months (AOR = 1.71, 95% CI: 1.40, 2.08) were more likely to have SAM than children age group 0–5 months.

As compared to children with birth order first, children with birth order between fourth and fifth were 1.24 times more likely to suffer from SAM (AOR = 1.24, 95% CI: 1.02, 1.53). Children who had a fever within 2 weeks were more likely to have SAM than those children who did not have a fever within 2 weeks (AOR = 1.31, 95% CI: 1.09, 1.57). Similarly, children who had anemia were 1.21 times more likely to experience SAM than those who were not anemic (AOR = 1.21, 95% CI: 1.07, 1.36). Children’s mothers’ age group between 25 and 34 years (AOR = 0.60, 95% CI: 0.50, 0.71) and 35–49 years (AOR = 0.49, 95% CI: 0.39, 0.62) had SAM decreased by 39.7 and 50.2%, respectively, as compared to children’s mothers’ age group of 15–24 years.

Children born to mothers who received ANC visits had a 19.7% lower risk of developing SAM than mothers who did not receive ANC visits (AOR = 0.80, 95% CI: 0.70, 0.91). Children born to rural residents were more likely to have SAM than children born from urban resident households (AOR = 2.98, 95% CI: 2.54, 3.49). Children living in households using solid fuel were 2.46 times more likely to suffer from SAM than children living in households using cleaner fuels (AOR = 2.46, 95% CI: 1.86, 3.26) (Table 2).

**Table 2:** Multivariable analysis for SAM among under-five children in Ethiopia, EDHS 2016.

Covariates	Odds ratio	SE	Z	*p*-value	[95% CI for AOR]	
Age of children						
0–5 months®						
6–11 months	1.52	0.16	4.16	0.001**	1.25	1.86
12–23 months	1.98	0.18	7.45	0.001**	1.65	2.37
24–59 months	1.71	0.17	5.33	0.001**	1.40	2.08
Birth order						
First®						
Second and third	1.06	0.096	0.62	0.533	0.89	1.27
Fourth and fifth	1.24	0.14	1.98	0.048*	1.02	1.54
Sixth and above	1.01	0.12	0.02	0.982	0.79	1.27
Fever						
No®						
Yes	1.31	0.12	2.90	0.004*	1.09	1.58
Anemia status						
Not anemic®						
Anemic	1.21	0.08	3.00	0.003*	1.07	1.36
Age of mother						
15–24 years®						
25–34 years	0.60	0.05	−5.78	0.001**	0.51	0.72
35–49 years	0.49		−6.02	0.001**	0.40	0.63
ANC visits						
No®						
Yes	0.80	0.05	−3.30	0.001**	0.705	0.92
Place of residence						
Urban®						
Rural	2.98	0.242	13.46	0.001**	2.54	3.49
Types of cooking fuel						
Cleaner fuel ®						
Solid fuel	2.46	0.35	6.30	0.001**	1.86	3.26
Constant	0.35	0.07	−5.34	0.001**	0.24	0.52

Significant at *p < 0.05; **p < 0.01; SE, standard error; ®, reference; Z, Wald test; AOR, adjusted odds ratio.

## Discussion

In this study, SAM among under-five children in Ethiopia was significantly associated with children’s age, higher birth order, mothers’ age, fever, anemic condition of children, mothers’ residence, type of cooking fuel, and ANC visit.

According to this EDHS report, the children’s age revealed a significant association with SAM. Those children in age groups 6–11 months, 12–23 months, and 24–59 months were more likely to develop SAM than children aged 0–5 months, implying that older children were more likely to suffer from SAM than younger. The result is in line with studies conducted in different parts of Ethiopia and Burkina Faso (5, 6, 14, 22, 23, 25, 31, 32). The possible reason for this finding was that almost all children were breastfed throughout their first 6 months of life, which might reduce the risk of SAM. Furthermore, the other reason, as a child becomes older, he or she is more likely to complete their vaccine, which reduces illness exposure. Another possible explanation is that caring habits tend to decline as children grow older and move from infancy to adulthood.

The birth order of children was significantly associated with SAM. Children with higher birth order had higher odds of SAM than those with lower birth order. The finding coincides with a study conducted in different parts of Ethiopia (5, 6, 33–35). The finding is in line with other countries in Nepal, South Asia, and India (1, 36, 37). This might be due to the fact that the birth order increases the number of children who may be affected by underfeeding. Moreover, parents give less attention to older children when they give birth to a new child who needs more attention and care than younger children. Another reason could be a lack of awareness of the spacing technique.

According to this EDHS report, febrile illness within 2 weeks was significantly associated with SAM. Children who had a fever within 2 weeks were more likely to suffer from SAM than those who did not have a fever within 2 weeks. This result concurs with a study conducted in Northwest Ethiopia (6, 38, 39). This might be due to febrile illness decreasing the appetite of the children.

Similarly, children who had anemia were more likely to experience SAM than those who did not have anemia. This result was in agreement with a study conducted in Bahr Dar city, Ethiopia, and Uganda (40, 41). Anemia is frequent in children with SAM, and it is caused by bone marrow hypoplasia, iron deficiency, vitamin B12 deficiency, vitamin A deficiency, and a lack of folate (42). Even though we did not look into these causes of anemia in our study, we believe they could explain the link between anemia and SAM in children.

Maternal healthcare utilization, particularly ANC use, was significantly associated with SAM. Children born from mothers who received ANC visits had a lower risk of developing SAM than children born from mothers who did not receive ANC visits. The finding is supported by the study conducted in Bahr Dar City, Ethiopia (6). Hence, mothers who visit ANC follow-up get child feeding and nutritional counseling.

The residence of the family was a significant predictor of SAM. As compared to children born in urban areas, the odds of SAM were higher in rural children. The result is similar to the study in Ethiopia (43, 44). These could be an integration of improved infant diets with improved sanitation and hygiene in urban residents that may improve the nutritional status of under-five children.

Maternal age was significantly associated with SAM. The children’s mothers’ age groups of 25–34 years and 35–49 years had lower rates of extreme acute malnutrition than children’s mothers aged 15–24 years. This finding was supported by a study conducted in Nepal, Arsi, Ethiopia, and Karnataka (1, 14, 45). This might be due to the fact that the older mothers paid great attention to children’s feeding practices.

Children from solid fuels user communities were more likely to develop SAM compared to children from cleaner fuels user communities. The result is in line with the study conducted in Ethiopia (5). The majority of Ethiopian households use solid fuels, such as wood, charcoal, and dung cake to meet their daily needs, which may decrease the nutritional contents of food.

### Strength and limitations

The strength of this study was that the research was conducted with nationally representative data, allowing the findings to be generalized. This study has its limitations. The cross-sectional data cannot be utilized to explore the causes of SAM outcomes or seasonal variations. Another limitation is that height measurements collected by lying down children under the age of 2 years during DHS data collection could be a source of human error. As a limitation, some variables, such as postnatal visit, Low Birth Weight (LBW), Intrauterine Growth Restriction (IUGR), complementary feeding, and intercurrent illness, were not included.

## Conclusion

The results from binary logistic regression showed that the age of children, birth order number, fever in the previous 2 weeks, anemia status, age of mother, ANC visits, place of residence, and type of cooking fuel were predictor variables contributing statistically significant effect in SAM among under-five children in Ethiopia. Older children age, higher birth order number, had a fever for 2 weeks, anemic children, living in rural, and solid fuel users suffered more from acute malnutrition while children of mothers age group of 25–34 years, 35–49 years, and having ANC visit reduced SAM from the significant predictors.

Ethiopia’s government recognizes that tackling the factors that contribute to child malnutrition is critical to long-term development. As a result, these findings highlight the need for existing policies to be revitalized and interventions to be implemented at the individual, community, and societal levels in Ethiopia to rescue and prevent children from contracting SAM. To prevent child SAM, impoverished households should be encouraged to participate in kitchen gardening to provide household food access, a healthy diet for their children, and promotion for mothers of young children.

## Data Availability

The original contributions presented in the study are included in the article/supplementary material, further inquiries can be directed to the corresponding author.

## Footnotes

^1^ www.dhsprogram.com
